# Revised “iRR6” model in intermediate‐1 risk myelofibrosis patients treated with ruxolitinib

**DOI:** 10.1002/cncr.70062

**Published:** 2025-08-21

**Authors:** Francesca Palandri, Filippo Branzanti, Massimiliano Bonifacio, Elena M. Elli, Erika Morsia, Mirko Farina, Mario Tiribelli, Giulia Benevolo, Eloise Beggiato, Bruno Martino, Giovanni Caocci, Novella Pugliese, Alessia Tieghi, Monica Crugnola, Gianni Binotto, Francesco Cavazzini, Elisabetta Abruzzese, Alessandro Isidori, Alessandra Dedola, Emilia Scalzulli, Andrea Duminuco, Luca Tosoni, Alda Strazimiri, Roberto M. Lemoli, Daniela Cilloni, Monica Bocchia, Fabrizio Pane, Chiara Sartor, Florian H. Heidel, Massimo Breccia, Giuseppe A. Palumbo, Andrew T. Kuykendall

**Affiliations:** ^1^ IRCCS Azienda Ospedaliero‐Universitaria di Bologna Istituto di Ematologia “Seràgnoli” Bologna Italy; ^2^ Dipartimento di Medicina Specialistica Diagnostica e Sperimentale Università di Bologna Bologna Italy; ^3^ Department of Engineering for Innovation Medicine Section of Innovation Biomedicine Hematology Area University of Verona Verona Italy; ^4^ Divisione di Ematologia e Unità Trapianto di Midollo IRCCS San Gerardo dei Tintori Monza Italy; ^5^ Hematology Unit Department of Clinical and Molecular Sciences DISCLIMO Università Politecnica delle Marche Ancona Italy; ^6^ Department of Clinical and Experimental Sciences Unit of Blood Diseases and Stem Cells Transplantation University of Brescia ASST Spedali Civili of Brescia Brescia Italy; ^7^ Division of Hematology and BMT Department of Medical Area University of Udine Udine Italy; ^8^ University Hematology Division Città della Salute e della Scienza Hospital Torino Italy; ^9^ Unit of Hematology Department of Oncology University of Torino Torino Italy; ^10^ Division of Hematology Reggio Calabria Azienda Ospedaliera “Bianchi Melacrino Morelli” Calabria Italy; ^11^ Ematologia Ospedale Businco Università degli studi di Cagliari Cagliari Italy; ^12^ Department of Clinical Medicine and Surgery Federico II University Medical School Naples Italy; ^13^ Department of Hematology Azienda USL‐IRCCS di Reggio Emilia Reggio Emilia Italy; ^14^ Haematology and BMT Centre Azienda Ospedaliero‐Universitaria di Parma Parma Italy; ^15^ Unit of Hematology and Clinical Immunology University of Padova Padova Italy; ^16^ Division of Hematology University of Ferrara Ferrara Italy; ^17^ Division of Hematology Ospedale S. Eugenio Roma Italy; ^18^ Hematology and Stem Cell Transplant Center AORMN Hospital Pesaro Italy; ^19^ Hematology Department of Translational and Precision Medicine Az. Policlinico Umberto I‐Sapienza University Rome Italy; ^20^ UO Ematologia AOU Policlinico “G. Rodolico”‐San Marco Catania Italy; ^21^ Department of Internal Medicine (DiMI) Clinic of Hematology University of Genoa Genoa Italy; ^22^ IRCCS Policlinico San Martino Genova Italy; ^23^ Department of Clinical and Biological Sciences University of Turin Turin Italy; ^24^ Hematology Unit Azienda Ospedaliera Universitaria Senese University of Siena Siena Italy; ^25^ Hematology Hemostasis Oncology and Stem Cell Transplantation Hannover Medical School (MHH) Hannover Germany; ^26^ Department of Scienze Mediche Chirurgiche e Tecnologie Avanzate “G.F. Ingrassia” University of Catania Catania Italy; ^27^ Department of Malignant Hematology H. Lee Moffitt Cancer Center Tampa Florida USA

**Keywords:** intermediate‐1, post‐ET myelofibrosis, post‐PV myelofibrosis, primary myelofibrosis, ruxolitinib, survival score

## Abstract

**Background:**

The response to ruxolitinib after 6 months (RR6) model allows early identification of ruxolitinib‐treated myelofibrosis (MF) patients with poorer overall survival (OS); however, it is less applicable to lower‐risk patients.

**Methods:**

To further explore this, the authors performed a subanalysis of the “RUX‐MF” study (NCT06516406) with an aim to validate the RR6 and to develop a score specific for intermediate‐1 DIPSS/MYSEC‐PM risk patients.

**Results:**

Among the 776 evaluable patients, 34.4%, 47.8%, and 17.8% were at low, intermediate, and high RR6 risk, with 5‐year OS of 64.1%, 51.8%, and 44.5%, respectively (*p* < .001). In the 428 intermediate‐1 patients, the RR6 model did not discriminate between intermediate and low‐risk patients (5‐year OS: 74.4% vs. 72.0%, *p* = .24). The intermediate‐1 specific RR6 (iRR6) model was therefore developed by incorporating new variables: underdosed ruxolitinib with respect to platelet count at one or more time points (hazard ratio [HR], 3.91; *p* < .001), absence of palpable spleen reduction by ≥50% at 6 months (HR, 1.45; *p* = .02), and red blood cell transfusion requirement at all time points (HR, 1.85; *p* = .01). The iRR6 model stratified patients into three risk categories: low (score 0, 20.3%), intermediate (score 1–2, 45.8%), and high‐risk (score >2, 33.9%), with 5‐year OS of 84.8%, 76.4%, and 56.6%, respectively (*p* < .0001). The iRR6 model was validated in a cohort of 95 intermediate‐1 risk patients from the Moffitt Cancer Center, yielding stratification into the same three risk categories, with 5‐year OS of 83.3% (low‐risk), 71.7% (intermediate‐risk), and 54.5% (high‐risk) (*p* = .01).

**Conclusions:**

The iRR6 model provides a more refined tool for the identification of intermediate‐1 MF patients who may benefit from early therapy shift.

## Abstract 2

The RR6 model allows for early identification of ruxolitinib‐treated myelofibrosis patients at higher risk for poorer overall survival but is less applicable to lower‐risk patients. A subanalysis of the Italian “RUX‐M” study, validated by a cohort from the Moffitt Cancer Center, led to the development of the intermediate‐1 specific RR6 (iRR6) model, which is specific for intermediate‐1 risk patients and may complement the RR6 score, offering a more granular risk stratification in patients with less advanced disease.

## INTRODUCTION

Myelofibrosis (MF) is a Philadelphia‐negative chronic myeloproliferative neoplasm (MPN) that can be primary (PMF) or secondary to polycythemia vera (PPV‐MF) or essential thrombocythemia (PET‐MF). It is characterized by progressive bone marrow fibrosis, splenomegaly, systemic symptoms, elevated risk of leukemic transformation, and reduced overall survival.^1^

In MF patients presenting splenomegaly and/or symptoms, Janus kinase (*JAK*) inhibitors are the most employed first‐line therapies. Ruxolitinib was the first‐in‐class *JAK1/2* inhibitor and has been shown to improve resolution of splenomegaly and symptoms in a significant proportion of patients, thereby prolonging life.^2^ However, approximately 50% of patients do not have a satisfactory response to ruxolitinib and approximately 50% of patients who initially respond experience disease re‐expansion over time.^2^

Consequently, approximately 70% of patients discontinue ruxolitinib after 5 years.^3^ In recent years, other *JAK2* inhibitors have become, or are about to become, available in clinical practice. These include fedratinib, a *JAK2*, *FLT3*, *BRD4* inhibitor that shares with ruxolitinib a discrete hematological toxicity and is effective in the control of splenomegaly in both first‐ and second‐line settings^4^; pacritinib, now usable in patients with severe thrombocytopenia^5^; and momelotinib, intended for patients with moderate to severe anemia.^6^, ^7^

Notwithstanding the advent of these novel therapeutic options and the multitude of ongoing clinical trials investigating drugs with disparate mechanisms of action, whether administered as monotherapy or in combination with *JAK2* inhibitors, the only truly curative therapy for MF remains allogeneic stem cell transplantation (ASCT).^8^

ASCT is performed on patients at higher risk of early mortality due to MF. These patients are identified using a variety of prognostic models, namely the International Prognostic Scoring System (IPSS) and its dynamic variant (DIPSS/DIPSS‐plus), as well as the mutation and karyotype‐enhanced IPSS (MIPSS70 and variants) for patients with PMF^9^, ^10^, ^11^, ^12^, ^13^, ^14^ and the myelofibrosis secondary to PV and ET‐prognostic model (MYSEC‐PM) and for those with PPV/PET‐MF.^15^

Identifying the optimal timing for transitioning from *JAK2* inhibitor therapy (primarily ruxolitinib) to transplantation or other alternative therapy/clinical trial remains a challenging and contentious issue in clinical practice. A delay in allogeneic transplantation in higher‐risk categories may result in a significant decline in transplant performance and patient outcomes.^16^

A recently developed model, the response to ruxolitinib after 6 months (RR6), has been created for the early identification of ruxolitinib‐treated patients who are projected to have a reduced survival. This facilitates their early selection for a therapeutic switch, particularly in relation to the possibility of early transplantation.^17^

Previous reports have validated the RR6 model in other MF cohorts.^18^, ^19^ Nevertheless, the ability to differentiate between intermediate and low risk patients was limited. Moreover, the extent to which this score can be applied to patients with intermediate‐1 risk remains unclear. Patients with intermediate‐1 risk MF are typically not considered for allogeneic stem cell transplantation and often present with less severe disease than those at higher risk, yet still experience notable splenomegaly and symptoms.^20^, ^21^ In these patients, ruxolitinib has demonstrated efficacy in managing symptoms and reducing splenomegaly, although a subset may exhibit suboptimal responses or disease progression, underscoring the necessity for further risk assessment models.^20^

## MATERIALS AND METHODS

### Patients’ population and study overview

At the time of data cutoff for the current analysis (February 2024), the retrospective study “RUX‐MF” collected 1055 MF patients, treated with ruxolitinib outside clinical trials, that were used as training cohort to validate the RR6 model.

Inclusion criteria in this subanalysis consisted of ≥6 months of follow‐up after initiation of ruxolitinib and available information on complete blood count, red blood cell (RBC) transfusion requirements, spleen length by palpation and ruxolitinib dose at baseline, and after 3 and 6 months of ruxolitinib therapy. At initiation of ruxolitinib, all patients had platelet count >50 × 10^9^/L and spleen palpable at ≥5 cm below the costal margin (BCM).

Subsequently, we focused on intermediate‐1 risk patients, to evaluate early predictors of worse overall survival in this specific population. A validation cohort comprising 95 patients with intermediate‐1 risk MF treated with ruxolitinib at the Moffitt Cancer Center (Florida) was also employed.

### Definitions

Diagnoses of PMF and PPV/PET‐MF were made according to 2016 World Health Organization criteria and International Working Group on Myelofibrosis Research and Treatment (IWG‐MRT) criteria, respectively.^22^, ^23^

The risk category was assessed at the time patients started on ruxolitinib according to the DIPSS or MYSEC‐PM, for primary MF and secondary MF, respectively.^10^, ^15^ Histologic examination was performed at local institutions; fibrosis was graded according to the European Consensus Grading System.^24^ Unfavorable karyotype was categorized as previously described.^11^ High molecular risk (HMR) pathogenetic mutations were defined as those including *ASXL1*, *SRSF2*, *EZH2*, *IDH1*, and *IDH2*, *U2AF1*.^12^ Anemia was defined according to Common Terminology Criteria for Adverse Events.^25^

MF‐related symptoms were assessed using the 10‐item Myeloproliferative Neoplasm Symptom Assessment Form Total Symptom Score (MPN10‐TSS).^26^ Spleen and symptoms responses were routinely assessed by palpation and by periodical total symptom score (TSS) evaluation, according to 2013 IWG‐MRT criteria.^23^

### Ethical aspects

The RUX‐MF study (NCT06516406) was performed in accordance with the guidelines of the institutional review boards of the participating centers and the standards of the Helsinki Declaration. The promoter of this study was the IRCCS Azienda Ospedaliero‐Universitaria S. Orsola‐Malpighi, Bologna, which obtained approval from the Area Vasta Emilia Centro Ethics Committee (approval file number: 048/2022/Oss/AOUBo). The study was approved by the local ethics committee of participating centers (protocol code: RUX‐MF) and has no commercial support.

### Statistical analysis

Continuous variables are expressed as medians and ranges or means and standard deviations, whereas categorical variables are presented as frequencies and percentages. We used the Wilcoxon‐Mann‐Whitney rank‐sum test or the *t*‐test for comparisons between groups, and associations between categorical variables (2‐way tables) were tested using the Fisher exact test or the χ^2^ test, as appropriate.

Prognostic factors for survival in intermediate‐1 patients were identified using univariate and multivariable Cox proportional hazards model. Multivariable Cox analysis was conducted on variables with *p* values <0.05 at univariate analysis, to assess hazard ratio (HR). To avoid the issue of multicollinearity and to remove highly correlated predictors from the model, collinearity among variables was detected using the Pearson correlation test. Variables that were associated with other factors in univariate analysis were excluded from the multivariable analysis. The following variables were assessed: (1) hemoglobin (Hb) decrease between 6 months and baseline, adjusted for baseline transfusions; (2) acquisition of leukocytosis, defined as white blood cells (WBC) >25 × 10^9^/L at 6 months in subjects with WBC ≤25 × 10^9^/L at baseline; (3) worsening thrombocytopenia, considering the following platelet (PLT) count categories: ≥200 × 10^9^/L, 100 to 199 × 10^9^/L, 75 to 99 × 10^9^/L, 50 to 74 × 10^9^/L, and <50 × 10^9^/L between 6 months and baseline; (4) underdosed ruxolitinib as a categorical variable, considering individuals “treated with ruxolitinib underdosed according to prescribing indication based on PLT count” versus those “treated with correct prescribing dose at all time points (baseline, 3 months, and/or 6 months)”; (5) reduction by ≤50% of palpable spleen length at 6 months (SR50), including no SR50 at months 3 and 6 and no SR50 at month 6, after SR50 at month 3; and (6) RBC transfusion requirement, considering the following categories “RBC transfusions at two time points,” and “RBC transfusions at all time points (baseline, 3 months, and 6 months).”

These variables were selected based on their mechanistic relevance to disease biology and treatment response in ruxolitinib‐treated MF patients. In particular, changes in hemoglobin, leukocyte, and platelet counts over time were evaluated as indicators of disease evolution or therapy‐related cytopenias. The requirement for RBC transfusion was included as a marker for persistent anemia and bone marrow dysfunction, both of which are known to have an adverse impact on prognosis.^27^ Underdosing of ruxolitinib relative to platelet count was tested in place of absolute dosing, to account for treatment intensity normalized to patient‐specific hematologic tolerance. This definition reflects adherence to the approved prescribing information and is more consistent with previous data showing that ruxolitinib underdosing may impact treatment outcomes.^28^ Finally, spleen response was assessed using a stricter cutoff (≥50% reduction by palpation), which was hypothesized to better reflect meaningful disease control in intermediate‐1 risk patients, who are more likely to achieve spleen shrinkage than high‐risk individuals. All variables were selected a priori based on clinical plausibility and were uniformly available across the study population.

Survival analysis comparing risk categories were performed using Kaplan–Meier curves, and differences were evaluated using log‐rank test. Overall survival (OS) was calculated from the date of ruxolitinib start, to either death, last contact, or ASCT.

Tests were two‐sided, and *p* values <.05 were considered significant. Analyses were performed using STATA/SE software version 18.0 (StataCorp).

## RESULTS

### Baseline characteristics of the entire cohort

The study included 776 patients from the RUX‐MF study cohort who met the inclusion criteria; 279 (36.0%) patients were excluded due to absence of palpable splenomegaly at baseline, ruxolitinib duration <6 months, or missing values required for RR6 validation (Figure S1)

### Overall cohort characteristics are summed up in Table 1

At the start of ruxolitinib, 55.2% of the patients were at intermediate‐1 risk. The median baseline hemoglobin level was 11.2 g/dL, with 132 (17.0%) patients having RBC transfusion requirement. The median platelet count was 265 × 10^9^/L (range: 56–1887), and 49.9% of the patients had splenomegaly palpable at ≥10 cm below the costal margin. Additionally, 58.0% of patients were highly symptomatic, with a TSS ≥20.

**TABLE 1:** Patient’s characteristics at RUX start.

	Total cohort (*n* = 776)	Intermediate‐1 (*n* = 428)
Age, years, median (range)	68.1 (24.7–92.0)	65.0 (24.7–88.2)
Age >65 years, No. (%)	485 (62.5)	217 (50.7)
Male sex, No. (%)	448 (57.7)	244 (57.0)
Primary MF, No. (%)	397 (51.2)	213 (49.8)
Post‐PV MF	216 (27.8)	135 (31.5)
Post‐ET MF	163 (21.0)	80 (18.7)
Driver mutation, No. (%)		
*JAK2*	593 (76.4)	338 (79.0)
*CALR*	96 (12.4)	52 (12.1)
*MPL*	17 (2.2)	7 (1.6)
Triple negative	40 (5.2)	17 (4.0)
Not available	30 (3.8)	14 (3.3)
RUX starting daily dose, No. (%)		
10–20 mg	282 (36.3)	188 (43.9)
30–40 mg	494 (63.7)	240 (56.1)
DIPSS or MYSEC‐PM score, No. (%)		
INT‐1	428 (55.2)	428 (100.0)
INT‐2/HIGH	348 (44.8)	0
HMR mutation, No. (%)	101/197 (51.3)	60/122 (49.2)
Hemoglobin, g/dL, median (range)	11.2 (5.0–18.3)	12.1 (6.9–18.3)
Hemoglobin <10 g/dL, No. (%)	272 (35.1)	24 (5.6)
RBC transfusions requirement, No. (%)	132 (17.0)	17 (4.0)
Platelet count, ×10^9^/L, median (range)	265 (56–1887)	283 (56–1632)
Platelet count <100 ×10^9^/L, No. (%)	68 (8.8)	32 (7.5)
White blood cell count, ×10^9^/L, median (range)	11.7 (1.1–155.0)	11.3 (1.1–78.9)
White blood cell count >25 ×10^9^/L, No. (%)	119 (15.3)	36 (8.4)
Peripheral blast count, mean ± SD	0.97 ± 1.61	113/417 (27.1%)
Blasts ≥1%, No. (%)	141/557 (25.3)	113 (26.4)
Spleen length BCM, median (range), cm	11 (5–35)	10 (5–35)
Spleen length BCM ≥10 cm, No. (%)	387 (49.9)	198 (46.3)
Total symptoms score, median (range)	20 (0–100)	20 (0–100)
Total symptoms score ≥20, No. (%)	450 (58.0)	223 (52.1)
Constitutional symptoms, No. (%)	574 (74.0)	143 (33.4)

Abbreviations: BCM, below costal margin; DIPSS, Dynamic International Prognostic Scoring System; ET, essential thrombocythemia; HMR, high molecular risk; INT, intermediate; MF, myelofibrosis; MYSEC‐PM, myelofibrosis secondary to PV and ET‐prognostic model; PV, polycythemia vera; RBC, red blood cells; RUX, ruxolitinib.

### Distribution of variables and validation of the RR6 in the entire cohort

The distribution of the RR6 variables was as follows: 33.4% of the patients received a ruxolitinib dose <20 mg twice daily at baseline, a proportion that increased to 64.2% at 3 months and 74.6% at 6 months. RBC transfusion requirement was recorded in 17% of patients at baseline, increasing to 34% at 3 months and remaining stable at 32.6% at 6 months. Approximately one‐third (31.7%) of patients failed to achieve a spleen reduction by at least 30% compared to baseline (SR30) at 3 months, with 37.6% not achieving SR30 at 6 months.

**FIGURE 1:**
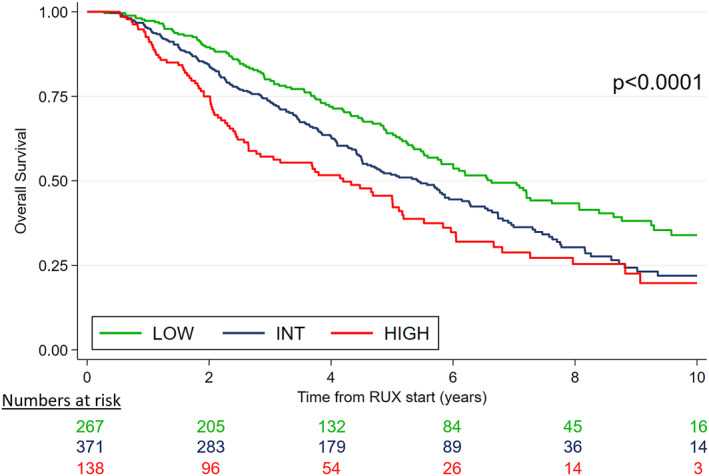
RR6 validation in the overall cohort. INT indicates intermediate; RR6, response to ruxolitinib after 6 months; RUX, ruxolitinib.

The RR6 model was first applied to the entire cohort. The RR6 model stratified patients into three risk groups: low‐risk (score 0, *n* = 267, 34.4%), intermediate‐risk (score 1–2, *n* = 371, 47.8%), and high‐risk (score >2, *n* = 138, 17.8%). The 5‐year OS rates were 64.1% for low‐risk patients, 51.8% for intermediate‐risk patients, and 44.5% for high‐risk patients (*p* < .0001), with statistically significant differences across all the risk categories (low vs. intermediate, *p* = .003; intermediate vs. high, *p* = .02; low vs. high, *p* = .0001) (Figure 1).

### Characteristics of the intermediate‐1 cohort

Of the total cohort, 428 patients were classified as intermediate‐1 risk according to DIPSS or MYSEC‐PM at ruxolitinib start (Table 1).

The distribution of the RR6 variables within the intermediate‐1 cohort was found to be consistent with that observed in the entire cohort: 57.0% of patients were receiving a ruxolitinib dose <20 mg twice daily at baseline, increasing to 69.4% at 3 months and 71.5% at 6 months. RBC transfusion requirement was recorded in 4.2% of patients at baseline, increasing sharply to 16.7% at 3 months, and 15.7% at 6 months. Approximately 41.4% of patients failed to achieve SR30 at 3 months, with 33.1% not reaching SR30 at 6 months (Table S1).

### Application of the RR6 model to the intermediate‐1 cohort

In univariate analysis, RBC transfusion requirements at all time points (HR, 2.65 [95% confidence interval (CI), 1.30–5.40], *p* = .007) and RBC transfusion requirement at 3 and 6 months (HR, 1.55 [95% CI, 1.10–2.17], *p* = 0.011) were significantly associated with a worse OS, whereas ruxolitinib dose <20 twice daily (HR, 1.22 [95% CI, 0.73–1.44], *p* = .50) and failure to achieve SR30 at 3 and/or 6 months (HR, 1.35 [95% CI, 0.90–1.52], *p* = .08), did not correlate with a worse prognosis. In multivariate analysis, only RBC transfusion requirement at all time points remained significantly associated with OS (HR, 2.10 [95% CI, 1.10–4.40], *p* = .05). Ruxolitinib dose <20 mg twice daily at all time points (HR, 1.10 [95% CI, 0.80–1.52], *p* = .49) and failure to achieve SR30 at 3 and/or 6 months (HR, 1.42 [95% CI, 0.98–1.99], *p* = .06) did not show significant predictive value for survival in this specific cohort (Figure S2).

**FIGURE 2:**
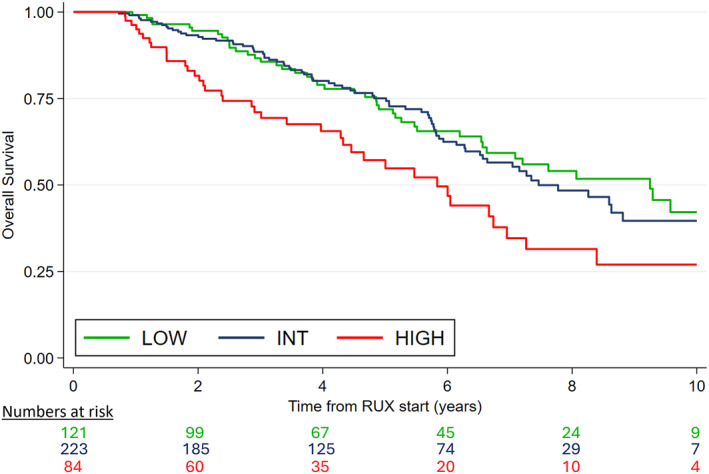
RR6 validation in intermediate‐1 risk patients. INT indicates intermediate; RR6, response to ruxolitinib after 6 months; RUX, ruxolitinib.

When the RR6 model was applied to the intermediate‐1 cohort, the 5‐year OS was 74.4% in the low‐risk group (*n* = 121, 28.3%) and 72.0% in the intermediate‐risk group (*n* = 223, 52.1%), indicating no significant difference between the two categories (*p* = .24). However, the high‐risk group (*n* = 84, 19.6%) showed a significantly poorer 5‐year OS of 57.4% (vs. low‐risk, *p* = .004; vs. intermediate‐risk, *p* = .009) (Figure 2).

### Development of the intermediate‐1 RR6 score

#### Testing new variables

**FIGURE 3:**
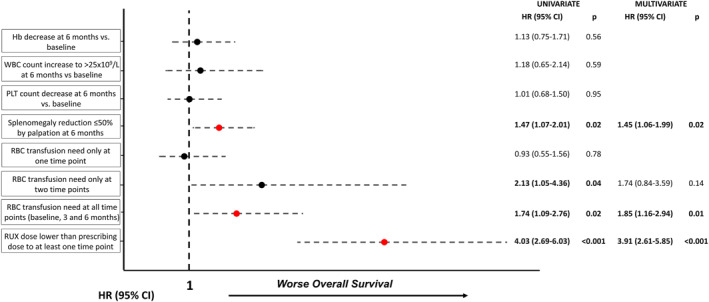
Variables associated with overall survival in the intermediate‐1 risk cohort. CI indicates confidence interval; Hb, hemoglobin; HR, hazard ratio; RBC, red blood cells; RUX, ruxolitinib; WBC, white blood cells.

New variables were tested in both univariate and multivariable analyses to improve risk stratification in intermediate‐1 patients (Figure 3). The following factors were found to be significant in multivariate analysis: underdosed ruxolitinib at any time point (HR, 3.91 [95% CI, 2.61–5.85], *p* < .001); absence of SR50 at 6 months (HR, 1.45 [95% CI, 1.06–1.99], *p* = .02); RBC transfusion requirement at all time points (HR, 1.85 [95% CI, 1.16–2.94], *p* = 0.01). RBC transfusion requirement at two time points was significant just in univariate analysis (univariate: HR, 2.13 [95% CI, 1.05–4.36], *p* = 0.05; multivariate: HR, 1.74 [95% CI, 0.84–3.59], *p* = 0.14).

Based on these findings, the intermediate‐1 RR6 (iRR6) model was developed, with the following point assignment: 2 points for underdosed ruxolitinib at any time points; 1.5 points for absence of SR50 at 6 months and for RBC transfusion requirement at all time points; and 1 point for RBC transfusion requirement at two time points.

The distribution of these variables in the intermediate‐1 cohort showed that: 40.0% received underdosed ruxolitinib at baseline, increasing at 45.6% and 41.8% of patients at 3 and 6 months respectively; 52.1% failed to achieve SR50 at 6 months; 18.7% required RBC transfusions at two time points, and 15.7% required transfusions at all time points (Table S1).

### Application of the iRR6 model in the RUX‐MF cohort

**FIGURE 4:**
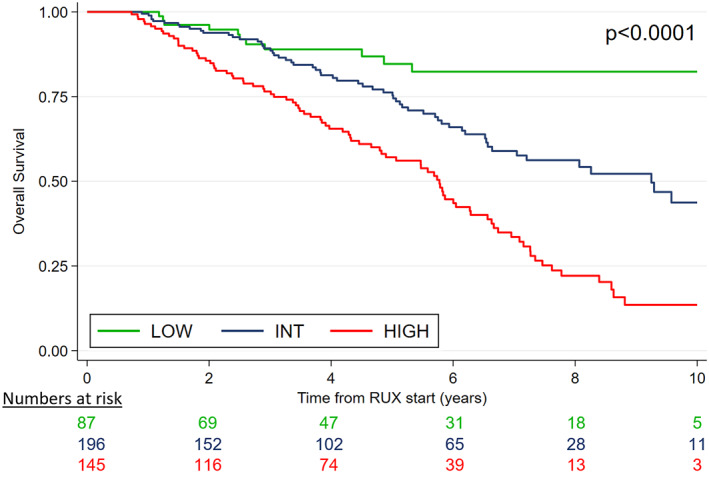
iRR6 model. INT indicates intermediate; iRR6, intermediate‐1 specific response to ruxolitinib after 6 months; RUX, ruxolitinib.

The iRR6 model was then applied to the intermediate‐1 cohort, effectively stratifying patients into three risk groups: low‐risk (score 0): 20.3% (*n =* 87) of patients with a 5‐year OS of 84.8% (median OS: not reached); intermediate‐risk (score 1–2): 45.8% (*n =* 196) of patients with a 5‐year OS of 76.4% (median OS: 9.24 years; 95% CI, 6.63–not reached); high‐risk (score >2): 33.9% of patients with a 5‐year OS of 56.6% (median OS: 5.77 years; 95% CI, 4.80–6.29) (*p* < .0001) (Figure 4).

To evaluate its performance across distinct prognostic classifications, we also applied the iRR6 model separately to PMF and SMF patients. In both the intermediate‐1 DIPSS‐defined PMF cohort and the MYSEC‐PM‐defined SMF cohort, the iRR6 model consistently identified three prognostic categories with significantly different overall survival (PMF: low‐risk, median OS: not reached; intermediate‐risk, median OS: 9.25 years; 95% CI, 5.93–not reached; high‐risk, median OS: 4.66 years; 95% CI, 3.42–5.77, *p* < .0001; SMF: low‐risk, median OS: not reached; intermediate‐risk: 9.58 years; 95% CI, 6.64–not reached; high‐risk, median OS: 6.29 years; 95% CI, 5.47–7.27, *p* < .0001) (Figure S3).

### Validation of the iRR6 model in the Moffitt Cancer Center cohort

To validate the iRR6 model, it was tested on a separate cohort of 95 intermediate‐1 patients from the Moffitt Cancer Center. The median age of this cohort was 63.4 years (range: 25.1–87.3) and 55.8% were male. Their baseline characteristics were comparable to those of the RUX‐MF cohort (Table S2).

The distribution of iRR6 variables in the Moffitt cohort was similar to the RUX‐MF cohort: 53.7% of patients received ruxolitinib underdosed at baseline, falling to 41.1% and 37.9% at 3 and 6 months, respectively; 55.8% failed to achieve SR50 at 6 months; 17.9% needed transfusion requirement at two time points, and 5.3% at all time points (Table S1).

The iRR6 model was successfully applied to the Moffitt cohort, yielding significant stratification into the same three risk groups: low‐risk: 16.8% (*n* = 16) of patients with a 5‐year OS of 83.3%; intermediate‐risk: 34.7% of patients (*n* = 33) with a 5‐year OS of 71.7%; high‐risk: 48.4% of patients (*n* = 46) with a 5‐year OS of 54.5% (*p* = .01) (Figure S4).

## DISCUSSION

The primary result of the present study is the validation of the RR6 model. In contrast to previous validations, in which intermediate and low‐risk patients exhibited nonsignificantly different survival rates, our cohort demonstrated that the RR6 effectively distinguished three categories with distinct survival expectations. This was likely attributable to the substantial patient sample size, which may have counterbalanced the inherent limitations of retrospective data collection.

The RR6 model was primarily developed to inform early therapeutic decisions in MF patients undergoing ruxolitinib treatment, with a specific focus on avoiding delays that could potentially compromise transplant outcomes. However, it was developed in a mixed cohort of higher‐risk patients, who are generally evaluated for transplantation, and intermediate‐1 risk patients, whose clinical presentation and treatment trajectory is heterogeneous.^29^

Optimal treatment of intermediate‐1 risk patients is a challenge. They, historically, were not included in the COMFORT studies and yet, they benefit from ruxolitinib therapy with higher‐response rates, less cytopenias, and lower discontinuation rates.^20^, ^30^ Furthermore, they constitute the majority of MF patients receiving ruxolitinib in a real‐life context. Finally, they are frequently not considered for transplantation, despite being younger patients who could potentially benefit more from the procedure. Therefore, it is crucial to identify the subset of intermediate‐1 risk patients who are unlikely to respond well so that we can optimize their therapy (or otherwise refer them for transplantation). In this study, we observed that the RR6 model, when applied specifically to intermediate‐1 patients, failed to distinguish between low‐ and intermediate‐risk patients. This highlights the need for more refined prognostic tools in this setting.^20^, ^21^

Failure to achieve an SR30 did not maintain a prognostic value in intermediate‐1 risk patients, who are instead better stratified using a more significant response cutoff (SR50). This confirms the better performance of ruxolitinib when used early in the disease course.^31^, ^32^, ^33^ Moreover, the nonuse of ruxolitinib maximum doses, which are often reserved for intermediate‐1 risk patients, was not significantly associated with survival. In contrast, failure to dose adequately with respect to platelet count confirmed a significant prognostic impact. Underdosing of ruxolitinib, often due to concerns over anemia, has been reported in previous studies in a substantial fraction of patients.^34^ Notably, the underdosing of ruxolitinib was associated not only with reduced response rates but also with poorer survival outcomes.^35^ Overall, these results underscore the significance of maintaining optimal dosing to ensure the greatest therapeutic effect and most favorable patient outcomes, particularly for intermediate‐1 risk patients, who could benefit most from this dosing strategy, with lesser toxicity.^36^, ^37^, ^38^, ^39^

The limitations of this study are acknowledged, including its retrospective nature, the suboptimal reliability of palpation for the assessment of spleen response, and the lack of information on patient adherence to ruxolitinib. Another limitation of this study is the incomplete availability of molecular data. As the RUX‐MF registry included patients treated from 2013 onward, next‐generation sequencing was not systematically implemented in clinical workflows at that time and was performed in only a subset of cases. Therefore, risk stratification at baseline was conducted using DIPSS or MYSEC‐PM, in alignment with real‐world clinical practice. Although we acknowledge that current prognostic models increasingly incorporate molecular features, our analysis focused on dynamic projection after 6 months of therapy, aiming to identify early indicators of long‐term outcome in intermediate‐1 risk patients. Prospective studies with comprehensive genomic profiling may further enhance the integration between molecular and treatment‐based risk models.

Despite these limitations, the validation of the iRR6 model in an independent cohort from the Moffitt Cancer Center lends further support to the generalizability and reliability of the model. Furthermore, a high degree of similarity was observed in the distribution of iRR6 variables between the Moffitt cohort and the original RUX‐MF cohort, suggesting that the iRR6 model can be applied broadly to intermediate‐1 patients treated with ruxolitinib, regardless of geographic or institutional disparities.

The iRR6 score does not contradict the original RR6 model but rather integrates it. The RR6 score continues to serve as a valuable tool for identifying patients who are likely to experience poorer outcomes during ruxolitinib therapy and its use in clinical practice must be encouraged. The iRR6 score has been developed to complement it, offering a more granular risk stratification in patients with less advanced disease, with the potential to mitigate the risks associated with prolonged ineffective therapies and delayed transitions to alternative treatments in this setting.

Further investigation is required to determine whether and which early interventions may ultimately improve outcomes. This should be the subject of future studies.

## AUTHOR CONTRIBUTIONS

**Francesca Palandri**: Conceptualization, investigation, funding acquisition, writing–original draft, writing–review and editing, data curation, resources, and visualization. **Filippo Branzanti**: Conceptualization, writing–original draft, writing–review and editing, data curation, formal analysis, and visualization. **Massimiliano Bonifacio**: Conceptualization, investigation, visualization, writing–review and editing, and resources. **Elena M. Elli**: Conceptualization, investigation, visualization, writing–review and editing, and resources. **Erika Morsia**: Conceptualization, investigation, visualization, writing–review and editing, resources. **Mirko Farina**: Conceptualization, investigation, visualization, resources, and writing–review and editing. **Mario Tiribelli**: Conceptualization, investigation, visualization, writing–review and editing, and resources. **Giulia Benevolo**: Conceptualization, investigation, visualization, writing–review and editing, and resources. **Eloise Beggiato**: Investigation, writing–review and editing, and resources. **Bruno Martino**: Investigation, writing–review and editing, and resources. **Giovanni Caocci**: Investigation, writing–review and editing, and resources. **Novella Pugliese**: Investigation, writing–review and editing, and resources. **Alessia Tieghi**: Investigation, writing–review and editing, and resources. **Monica Crugnola**: Investigation, writing–review and editing, and resources. **Gianni Binotto**: Investigation, writing–review and editing, and resources. **Francesco Cavazzini**: Investigation, writing–review and editing, and resources. **Elisabetta Abruzzese**: Investigation, writing–review and editing, and resources. **Alessandro Isidori**: Investigation, writing–review and editing, and resources. **Alessandra Dedola**: Conceptualization, Investigation, visualization, writing–review and editing, and resources. **Emilia Scalzulli**: Investigation, writing–review and editing, and resources. **Andrea Duminuco**: Investigation, writing–review and editing, and resources. **Luca Tosoni**: Investigation, writing–review and editing, and resources. **Alda Strazimiri**: Investigation, writing–review and editing, and resources. **Roberto M. Lemoli**: Investigation, writing–review and editing, and resources. **Daniela Cilloni**: Investigation, writing–review and editing, and resources. **Monica Bocchia**: Investigation, writing–review and editing, and resources. **Fabrizio Pane**: Investigation, writing–review and editing, and resources. **Chiara Sartor**: Investigation, writing–review and editing, and resources. **Florian H. Heidel**: Visualization, Writing–review and editing. **Massimo Breccia**: Conceptualization, investigation, visualization, writing–review and editing, and resources. **Giuseppe A. Palumbo**: Conceptualization, investigation, visualization, writing–review and editing, and resources. **Andrew T. Kuykendall**: Conceptualization, investigation, visualization, writing–review and editing, and resources.

## CONFLICT OF INTEREST STATEMENT

Elisabetta Abruzzese reports consulting fees from Ascentage Pharma, Bristol‐Myers Squibb, GlaxoSmithKline, Incyte, Instituto Científico Pfizer, and Novartis. Giulia Benevolo reports consulting fees from Bristol‐Myers Squibb and GlaxoSmithKline; fees for professional activities from AOP Health; and honoraria from Novartis, Janssen, Amgen, and Takeda. Gianni Binotto reports honoraria from Novartis, Incyte, Bristol‐Myers Squibb‐Celgene, and Pfizer. Monica Bocchia reports consulting fees from Incyte and Novartis; and travel fees from BeiGene USA, Inc. Massimo Breccia reports honoraria from Novartis, Bristol‐Myers Squibb, Pfizer, and Incyte. Monica Crugnola reports honoraria from Novartis and Amgen. Andrea Duminuco reports consulting fees from A.O.U. Policlinico “G. Rodolico‐San Marco.” Florian H. Heidel reports consulting fees from AbbVie, AOP Orphan Pharmaceuticals, Bristol‐Myers Squibb, CTI Biopharma, GlaxoSmithKline, Janssen Pharmaceuticals, Merck, Novartis, Prelude Therapeutics, and Silence Pharmaceuticals. Andrew T. Kuykendall has received honorarium and/or consulting fees from Incyte, Protagonist, Kartos Therapeutics, PharmaEssentia, AbbVie, Imago Biosciences, Karyopharm Therapeutics Inc, PharmaEssentia, Blueprint Medicines Corporation, Constellation, CTI Biopharma, Novartis, and Sierra Oncology; and grant and/or contract funding from Protagonist Therapeutics, Inc, Bristol‐Myers Squibb, Constellation, Geron Corporation, Janssen Pharmaceuticals, Novartis, and Sierra Oncology. Roberto M. Lemoli reports honoraria from Jazz, Pfizer, AbbVie, Bristol‐Myers Squibb, Sanofi, and StemLine. Francesca Palandri participated in the speakers bureau and advisory board of Novartis, Bristol‐Myers Squibb, AOP Health, Sierra Oncology, Incyte, Telios, AbbVie, Constellation‐Morphosys, Sobi and GlaxoSmithKline, Sanofi, and Takeda Oncology. Giuseppe A. Palumbo reports consultancy and honoraria from AbbVie, AOP, AstraZeneca, Bristol‐Myers Squibb, Incyte, GlaxoSmithKline, Morphosys, and Novartis; and travel fees from AbbVie, AstraZeneca, BeiGene, Ltd, Janssen Biotech, Sobi, and Stemline Therapeutics Inc. Fabrizio Pane reports honoraria from Incyte, Novartis, Jazz, Bristol‐Myers Squibb‐Celgene, Amgen, and Gilead. Mario Tiribelli reports honoraria from and has served on speakers’ bureaus for Novartis, Bristol‐Myers Squibb, Pfizer, and Incyte. Massimiliano Bonifacio reports honoraria from Novartis, Bristol‐Myers Squibb, Pfizer, and Incyte, Amgen, Ascentage Pharma, Blueprint Medicines, Clinigen, and Glaxo‐Smith Kline. The other authors declare no conflicts of interest.

## Supporting information

Supplementary Material

## Data Availability

The data that support the findings of this study are available from the corresponding author on reasonable request to the corresponding author (francesca.palandri@unibo.it) at the following https://zenodo.org/records/14017637.

## References

[1] Passamonti F , Mora B . Myelofibrosis. Blood. 2023;141(16):1954‐1970. doi:10.1182/BLOOD.2022017423 36416738 PMC10646775

[2] Al‐Ali HK , Griesshammer M , le Coutre P , et al. Safety and efficacy of ruxolitinib in an open‐label, multicenter, single‐arm phase 3b expanded‐access study in patients with myelofibrosis: a snapshot of 1144 patients in the JUMP trial. Haematologica. 2016;101(9):1065‐1073. doi:10.3324/HAEMATOL.2016.143677 27247324 PMC5060023

[3] Palandri F , Breccia M , Bonifacio M , et al. Life after ruxolitinib: reasons for discontinuation, impact of disease phase, and outcomes in 218 patients with myelofibrosis. Cancer. 2020;126(6):1243‐1252. doi:10.1002/CNCR.32664 31860137

[4] Harrison CN , Schaap N , Vannucchi AM , et al. Janus kinase‐2 inhibitor fedratinib in patients with myelofibrosis previously treated with ruxolitinib (JAKARTA‐2): a single‐arm, open‐label, non‐randomised, phase 2, multicentre study. Lancet Haematol. 2017;4(7):e317‐e324. doi:10.1016/S2352-3026(17)30088-1 28602585 PMC8207822

[5] Mascarenhas J . Pacritinib for the treatment of patients with myelofibrosis and thrombocytopenia. Expert Rev Hematol. 2022;15(8):671‐684. doi:10.1080/17474086.2022.2112565 35983661

[6] Mesa RA , Kiladjian JJ , Catalano JV , et al. SIMPLIFY‐1: a phase III randomized trial of momelotinib versus ruxolitinib in Janus kinase inhibitor‐naïve patients with myelofibrosis. J Clin Oncol. 2017;35(34):3844‐3850. doi:10.1200/JCO.2017.73.4418 28930494 PMC6553796

[7] Harrison CN , Vannucchi AM , Platzbecker U , et al. Momelotinib versus best available therapy in patients with myelofibrosis previously treated with ruxolitinib (SIMPLIFY 2): a randomised, open‐label, phase 3 trial. Lancet Haematol. 2018;5(2):e73‐e81. doi:10.1016/S2352-3026(17)30237-5 29275119

[8] Devlin R , Gupta V . Myelofibrosis: to transplant or not to transplant? Hematology. 2016;2016(1):543‐551. doi:10.1182/ASHEDUCATION-2016.1.543 27913527 PMC6142493

[9] Cervantes F , Dupriez B , Pereira A , et al. New prognostic scoring system for primary myelofibrosis based on a study of the International Working Group for Myelofibrosis Research and Treatment. Blood. 2009;113(13):2895‐2901. doi:10.1182/BLOOD-2008-07-170449 18988864

[10] Passamonti F , Cervantes F , Vannucchi AM , et al. A dynamic prognostic model to predict survival in primary myelofibrosis: a study by the IWG‐MRT (International Working Group for Myeloproliferative Neoplasms Research and Treatment). Blood. 2010;115(9):1703‐1708. doi:10.1182/BLOOD-2009-09-245837 20008785

[11] Gangat N , Caramazza D , Vaidya R , et al. DIPSS plus: a refined Dynamic International Prognostic Scoring System for primary myelofibrosis that incorporates prognostic information from karyotype, platelet count, and transfusion status. J Clin Oncol. 2011;29(4):392‐397. doi:10.1200/JCO.2010.32.2446 21149668

[12] Tefferi A , Guglielmelli P , Lasho TL , et al. MIPSS70+ version 2.0: mutation and karyotype‐enhanced international prognostic scoring system for primary myelofibrosis. J Clin Oncol. 2018;36(17):1769‐1770. doi:10.1200/JCO.2018.78.9867 29708808

[13] Guglielmelli P , Lasho TL , Rotunno G , et al. MIPSS70: mutation‐enhanced international prognostic score system for transplantation‐age patients with primary myelofibrosis. J Clin Oncol. 2018;36(4):310‐318. doi:10.1200/JCO.2017.76.4886 29226763

[14] Vannucchi AM , Lasho TL , Guglielmelli P , et al. Mutations and prognosis in primary myelofibrosis. Leukemia. 2013;27(9):1861‐1869. doi:10.1038/leu.2013.119 23619563

[15] Passamonti F , Giorgino T , Mora B , et al. A clinical‐molecular prognostic model to predict survival in patients with post polycythemia vera and post essential thrombocythemia myelofibrosis. Leukemia. 2017;31(12):2726‐2731. doi:10.1038/leu.2017.169 28561069

[16] Gagelmann N , Mora B , Branzanti F , et al. Personalized transplant decision making for myelofibrosis in the era of molecular genetics and JAK inhibition. Blood. 2024;144(suppl 1):245. doi:10.1182/BLOOD-2024-210804 39023869

[17] Maffioli M , Mora B , Ball S , et al. A prognostic model to predict survival after 6 months of ruxolitinib in patients with myelofibrosis. Blood Adv. 2022;6(6):1855‐1864. doi:10.1182/BLOODADVANCES.2021006889 35130339 PMC8941454

[18] Scalzulli E , Ielo C , Luise C , et al. RR6 prognostic model provides information about survival for myelofibrosis treated with ruxolitinib: validation in a real‐life cohort. Blood Adv. 2022;6(15):4424‐4426. doi:10.1182/BLOODADVANCES.2022008158 35737865 PMC9636318

[19] Duminuco A , Nardo A , Garibaldi B , et al. Prediction of survival and prognosis migration from gold‐standard scores in myelofibrosis patients treated with ruxolitinib applying the RR6 prognostic model in a monocentric real‐life setting. J Clin Med. 2022;11(24):7418. doi:10.3390/JCM11247418 36556033 PMC9783796

[20] Palandri F , Elli EM , Morsia E , et al. Clinical outcomes of ruxolitinib treatment in 595 intermediate‐1 risk patients with myelofibrosis: the RUX‐MF real‐world study. Cancer. 2024;130(24):4257‐4266. doi:10.1002/CNCR.35489 39078647 PMC11585342

[21] Palandri F , Tiribelli M , Benevolo G , et al. Efficacy and safety of ruxolitinib in intermediate‐1 IPSS risk myelofibrosis patients: results from an independent study. Hematol Oncol. 2018;36(1):285‐290. doi:10.1002/HON.2429 28512865

[22] Arber DA , Orazi A , Hasserjian R , et al. The 2016 revision to the World Health Organization classification of myeloid neoplasms and acute leukemia. Blood. 2016;127(20):2391‐2405. doi:10.1182/BLOOD-2016-03-643544 27069254

[23] Tefferi A , Cervantes F , Mesa R , et al. Revised response criteria for myelofibrosis: International Working Group‐Myeloproliferative Neoplasms Research and Treatment (IWG‐MRT) and European LeukemiaNet (ELN) consensus report. Blood. 2013;122(8):1395‐1398. doi:10.1182/BLOOD-2013-03-488098 23838352 PMC4828070

[24] Gianelli U , Vener C , Bossi A , et al. The European Consensus on grading of bone marrow fibrosis allows a better prognostication of patients with primary myelofibrosis. Mod Pathol. 2012;25(9):1193‐1202. doi:10.1038/modpathol.2012.87 22627739

[25] Cancer Institute N . Common Terminology Criteria for Adverse Events (CTCAE) Common Terminology Criteria for Adverse Events (CTCAE) v5.0. 2017. Accessed June 20, 2024. https://www.meddra.org/

[26] Emanuel RM , Dueck AC , Geyer HL , et al. Myeloproliferative neoplasm (MPN) symptom assessment form total symptom score: prospective international assessment of an abbreviated symptom burden scoring system among patients with MPNs. J Clin Oncol. 2012;30(33):4098‐4103. doi:10.1200/JCO.2012.42.3863 23071245 PMC4872304

[27] Nicolosi M , Mudireddy M , Lasho TL , et al. Sex and degree of severity influence the prognostic impact of anemia in primary myelofibrosis: analysis based on 1109 consecutive patients. Leukemia. 2018;32(5):1254‐1258. doi:10.1038/S41375-018-0028-X 29568091 PMC5940639

[28] Breccia M , Palandri F , Martelli M , et al. Dosing and clinical outcomes of ruxolitinib in patients with myelofibrosis in a real‐world setting: Interim results of the Italian observational study (ROMEI). Cancer. 2025;131(7):e35801. doi:10.1002/cncr.35801 40111826 PMC11925231

[29] Kröger N , Bacigalupo A , Barbui T , et al. Indication and management of allogeneic haematopoietic stem‐cell transplantation in myelofibrosis: updated recommendations by the EBMT/ELN International Working Group. Lancet Haematol. 2024;11(1):e62‐e74. doi:10.1016/S2352-3026(23)00305-8 38061384

[30] Hu S , Yang X , Lu Y , et al. Comparing the efficacy and safety of ruxolitinib in patients with lower and higher risk myelofibrosis: a multi‐center, single‐arm, exploratory and prospective study. Blood. 2024;144(suppl 1):1786. doi:10.1182/BLOOD-2024-205090

[31] Palandri F , Al‐Ali HK , Guglielmelli P , Zuurman MW , Sarkar R , Gupta V . Benefit of early ruxolitinib initiation regardless of fibrosis grade in patients with primary myelofibrosis: a post hoc analysis of the single‐arm phase 3b JUMP study. Cancers (Basel). 2023;15(10):2859. doi:10.3390/cancers15102859 37345196 PMC10216208

[32] Verstovsek S , Kiladjian JJ , Vannucchi AM , et al. Early intervention in myelofibrosis and impact on outcomes: a pooled analysis of the COMFORT‐I and COMFORT‐II studies. Cancer. 2023;129(11):1681‐1690. doi:10.1002/CNCR.34707 36840971

[33] Verstovsek S , Gotlib J , Mesa RA , et al. Long‐term survival in patients treated with ruxolitinib for myelofibrosis: COMFORT‐I and ‐II pooled analyses. J Hematol Oncol. 2017;10(1):156. doi:10.1186/S13045-017-0527-7 28962635 PMC5622445

[34] Palandri F , Breccia M , Mazzoni C , et al. Ruxolitinib in cytopenic myelofibrosis: response, toxicity, drug discontinuation, and outcome. Cancer. 2023;129(11):1704‐1713. doi:10.1002/CNCR.34722 36932983

[35] Breccia M , Celant S , Palandri F , et al. The impact of starting dose on overall survival in myelofibrosis patients treated with ruxolitinib: a prospective real‐world study on AIFA monitoring registries. Br J Haematol. 2024;206(1):172‐179. doi:10.1111/BJH.19812 39363576

[36] Pemmaraju N , Bose P , Rampal R , Gerds AT , Fleischman A , Verstovsek S . Ten years after ruxolitinib approval for myelofibrosis: a review of clinical efficacy. Leuk Lymphoma. 2023;64(6):1063‐1081. doi:10.1080/10428194.2023.2196593 37081809

[37] Rossetti JM , Choksi R , Suthar P , et al. An analysis of ruxolitinib dosing for myelofibrosis in real‐world practice. Blood. 2023;142(suppl 1):5186. doi:10.1182/BLOOD-2023-179402

[38] Talpaz M , Erickson‐Viitanen S , Hou K , Hamburg S , Baer MR . Evaluation of an alternative ruxolitinib dosing regimen in patients with myelofibrosis: an open‐label phase 2 study. J Hematol Oncol. 2018;11(1):101. doi:10.1186/S13045-018-0642-0 30086777 PMC6081850

[39] Cervantes F , Ross DM , Radinoff A , et al. Efficacy and safety of a novel dosing strategy for ruxolitinib in the treatment of patients with myelofibrosis and anemia: the REALISE phase 2 study. Leukemia. 2021;35(12):3455‐3465. doi:10.1038/S41375-021-01261-X 34017073 PMC8632662

