# Supplementary material for: Revised “iRR6” model in intermediate‐1 risk myelofibrosis patients treated with ruxolitinib
Source: Cancer. 2025 Aug 21;131(17):e70062. doi: 10.1002/cncr.70062 (PMC12369922; doi:10.1002/cncr.70062)
Supplement: Supplementary file 1 — Supplementary Material [file CNCR-131-e70062-s001.docx]

### Supplemental Figure 1: Patients’ disposition


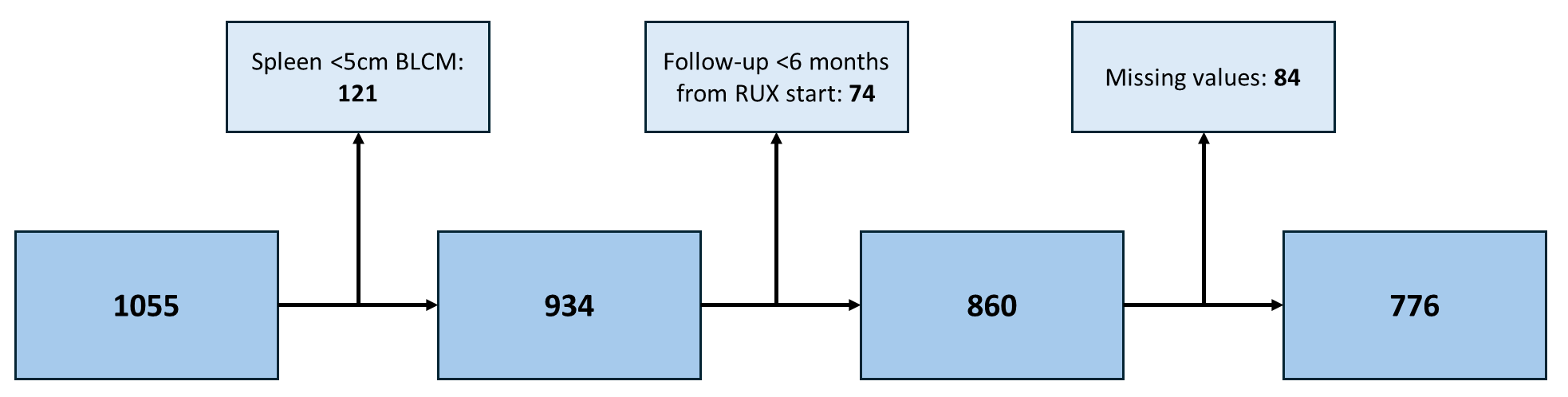


Supplemental Figure 1: BLCM, below left costal margin; RUX, ruxolitinib

### Supplemental Figure 2: Multivariable analysis of RR6 variables in Intermediate-1 risk patients


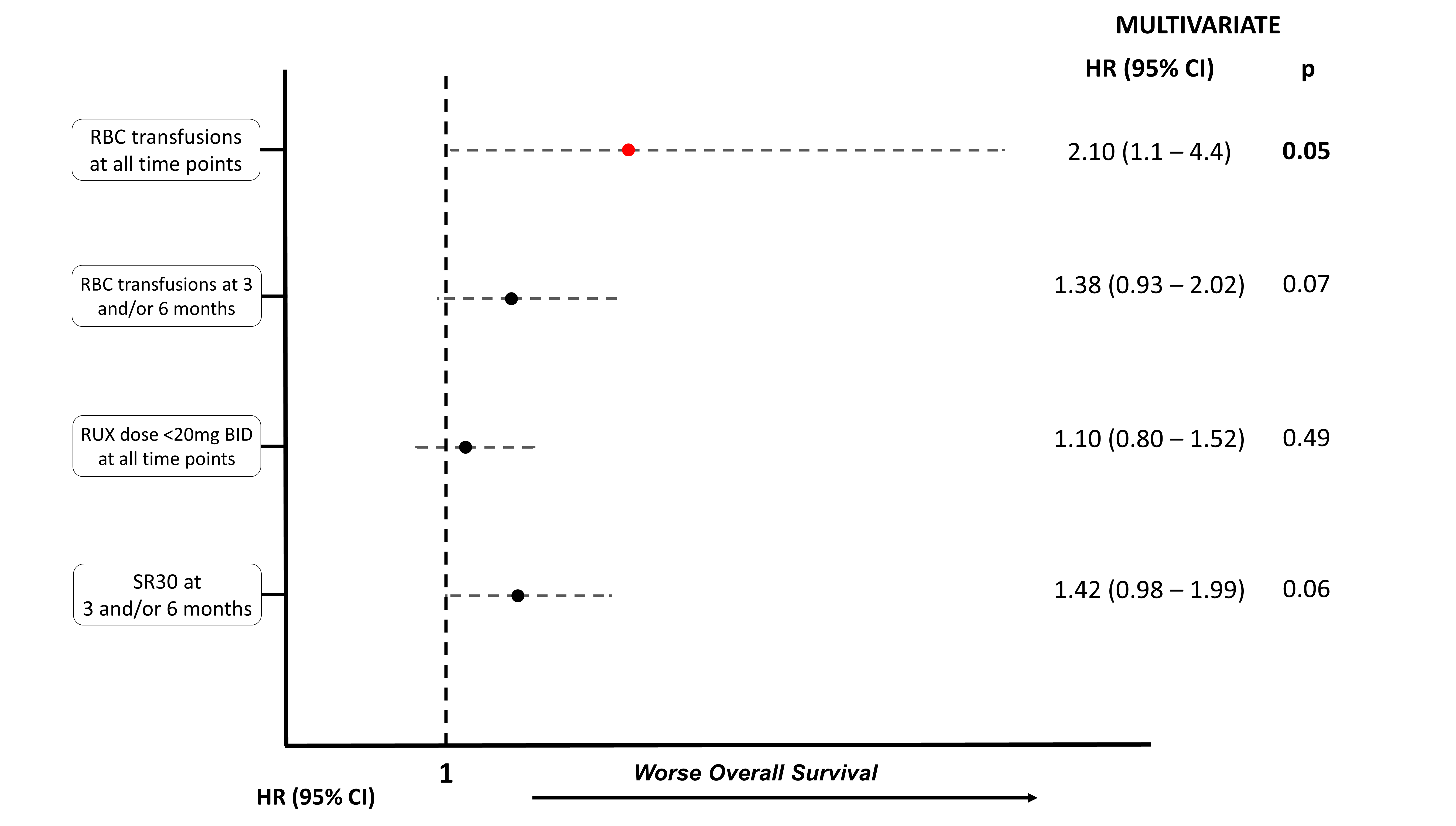


Supplemental Figure 2: RBC, Red Blood Cells; BID, Bis In Die; RUX, ruxolitinib; SR30, spleen reduction of at least 30% from baseline; HR, Hazard Ratio; CI, Confidence Interval.

### Supplemental Figure 3: iRR6 model application in intermediate-1 patients with Primary Myelofibrosis (a) and Secondary Myelofibrosis (b) separately


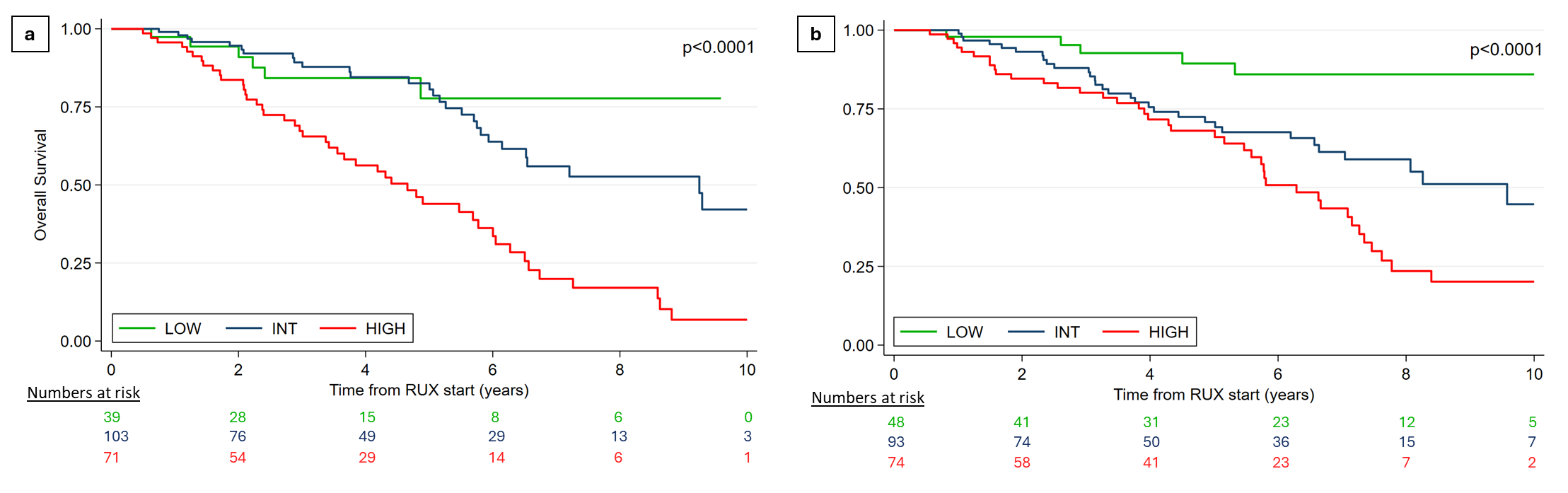


Supplemental Figure 3: RUX, ruxolitinib; INT, intermediate. (a) Intermediate-1 patients defined by DIPSS; (b) Intermediate-1 patients defined by MYSEC-PM.

### Supplemental Figure 4: Validation of iRR6 model on Moffitt Center Cohort


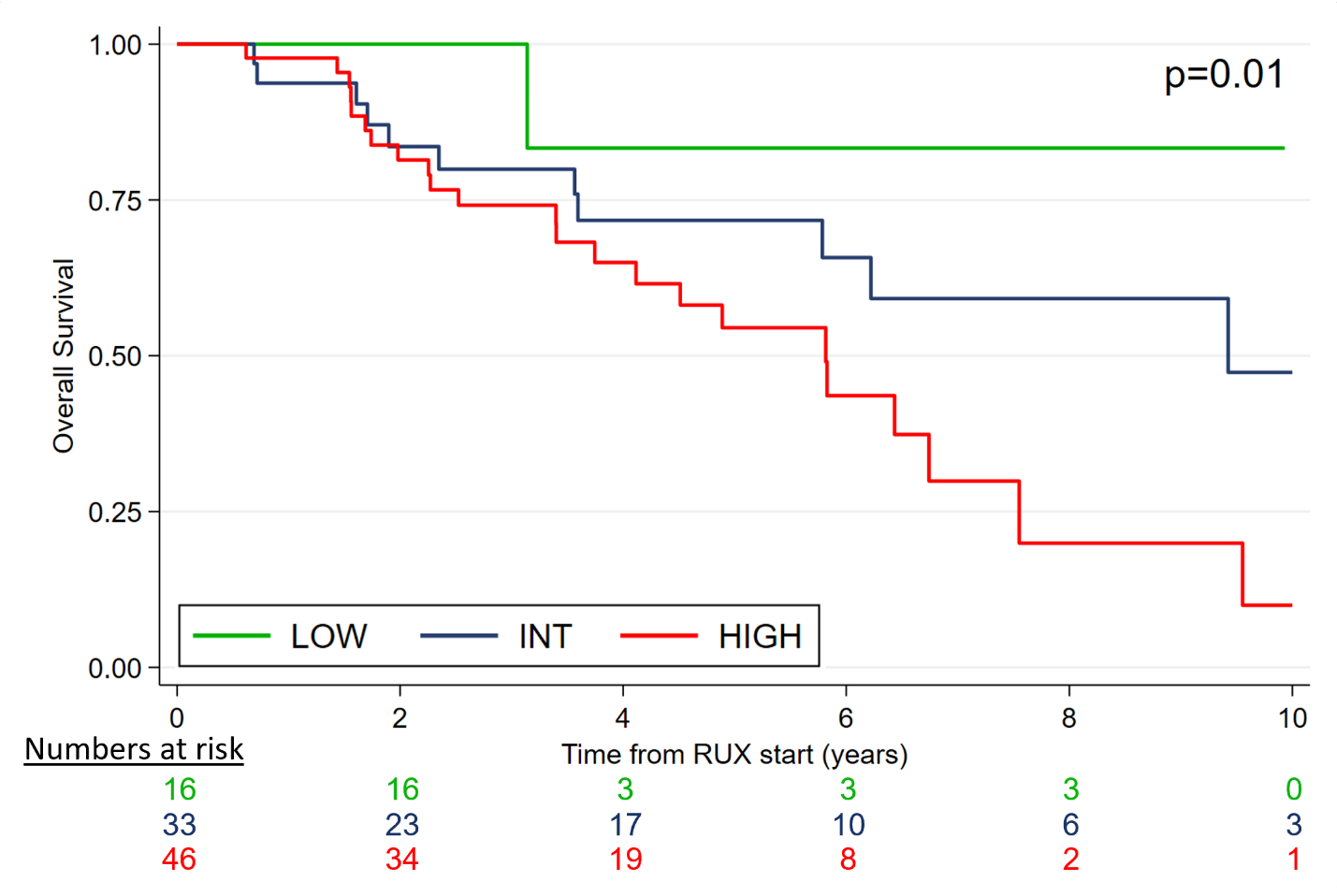


Supplemental Figure 4: RUX, ruxolitinib; INT, intermediate

### Supplemental Table 1: Distribution of variables across RR6/iRR6 models

|  | **Cohort** | **Variable** | **Baseline** | **3 months** | **6 months** | **All time points** |
| --- | --- | --- | --- | --- | --- | --- |
| RR6 | Overall Cohort  (RUX-MF) | RUX <20mg BID, % | 59.9% | 64.2% | 74.6% | 33.4% |
|  |  | RBC transfusions need, % | 17.0% | 34.0% | 32.6% | 13.3% |
|  |  | No SR30, % | n.a. | 43.4% | 37.6% | 31.7% |
| RR6 | INT-1 patients  (RUX-MF) | RUX <20mg BID, % | 57.0% | 69.4% | 71.5% | 52.8% |
|  |  | RBC transfusions need, % | 4.2% | 16.7% | 15.7% | 2.8% |
|  |  | No SR30, % | n.a. | 41.4% | 33.1% | 29.2% |
| iRR6 | INT-1 patients  (RUX-MF) | RUX underdosed, % | 40.0% | 45.6% | 41.8% | 22.2% |
|  |  | RBC transfusions need, % | 5.4% | 18.7% | 15.7% | 2.8% |
|  |  | No SR50, % | n.a. | 56.3% | 52.1% | 47.0% |
| iRR6 | INT-1 patients  (Moffitt Cancer Center) | RUX underdosed, % | 53.7% | 41.1% | 37.9% | 24.2% |
|  |  | RBC transfusions need, % | 5.3% | 17.9% | 15.8% | 5.3% |
|  |  | No SR50, % | n.a. | 50.5% | 55.8% | 35.8% |

Supplemental Table 1: INT, intermediate; RUX, ruxolitinib; BID, Bis In Die; RBC, Red Blood Cells; SR30, spleen reduction of at least 30% from baseline; SR50, spleen reduction of at least 50% from baseline

### Supplemental Table 2: overall Moffitt Center intermediate-1 cohort characteristics

|  | At ruxolitinib treatment initiation  (n. 95) |
| --- | --- |
| Age, years, median (range)  *Age >65 years, n. (%)* | 63.4 (25.1 – 87.3)  59 (62.1%) |
| Male sex, n. (%) | 53 (55.8%) |
| Ruxolitinib starting daily dose, n. (%),  *10-20 mg*  *30-40 mg* | 47 (49.5%)  48 (50.5%) |
| Transfusion pre-ruxolitinib start, n. (%) | 5 (5.3%) |
| Platelet count, x10^9^/L, median (range)  *Platelet count <100 x10^9^/L* | 224 (52 – 955)  9 (9.5%) |
| Spleen length below costal margin, median (range), cm  *Spleen length below costal margin ≥10 cm, n. (%)* | 8 (5 - 23)  41 (43.2%) |
